# Spatiotemporal Changes of Calcitonin Gene-Related Peptide Innervation in Spinal Fusion

**DOI:** 10.1155/2016/5872860

**Published:** 2016-11-21

**Authors:** Xiao-Yi Zhou, Xi-Ming Xu, Sui-Yi Wu, Fei Wang, Yi-Lin Yang, Ming Li, Xian-Zhao Wei

**Affiliations:** ^1^Department of Orthopaedic Surgery, Changhai Hospital, Second Military Medical University, Shanghai, China; ^2^Department of Orthopaedic Surgery, Changzheng Hospital, Second Military Medical University, Shanghai, China; ^3^Faculty of Naval Medicine, Second Military Medical University, Shanghai, China

## Abstract

Few studies have investigated the role calcitonin gene-related peptide (CGRP) plays in the process of spinal fusion. The aim of the present study is to observe the temporal and spatial changes of CGRP induced by experimental fusion surgery in rats and elucidate the role of CGRP in spinal fusion. Male Sprague-Dawley rats were used in the study and the specimens were collected on the 7th, 14th, 21st, and 28th day, respectively. Then, histological and immunohistochemical analysis were applied to evaluate the fusion mass and spatiotemporal changes of CGRP chronologically. The results demonstrated that density of CGRP reached peak on the 21st day after surgery and most of the CGRP expression located surrounding the interface of allograft and fibrous tissue where the cells differentiate into osteoblasts, indicating that CGRP might be involved in the process of bone formation and absorption.

## 1. Introduction

The process of bone regeneration and remodeling has been extensively studied, but the mechanism of bone repair after spinal fusion surgery is poorly understood. Systemically, bone repair is affected by hormones such as parathyroid hormone and insulin-like growth factor [1, 2]. Locally, bone modelling is regulated by regional factors, for instance, various growth factors and cytokines [3–5]. In addition, increasing evidences reveal that the process of bone regeneration and remodeling is closely related to nerve fiber innervation [6]. Hence, autonomic, sensory, and opioid neuropeptides have been investigated and found to regulate bone metabolism through specific receptors.

Calcitonin gene-related peptide (CGRP), a potent vasodilator, is mainly synthesized in small/medium sized sensory neurons in dorsal root ganglia and secreted by central and peripheral nervous system [7]. CGRP is reported to act as a regulatory factor in bone metabolism [8] and to be involved in angiogenesis [9] during bone repair. The presence of CGRP-positive nerve fibers would increase callus formation and bone repair during spinal fusion [10]. Locating in osteoblasts and osteoclasts, CGRP receptors were found to affect bone regeneration and remodeling by several studies. Meanwhile, decreased CGRP-positive sensory nerve innervation was observed in nonunion of bone fracture healing, indicating the important role of CGRP during bone regeneration [11].

Since callus formation and bone homeostasis are closely accompanied by CGRP expression and few studies explored the effects of CGRP in spinal fusion, the present study is aimed at (1) investigating the spatiotemporal changes of CGRP during spinal fusion and (2) trying to elucidate the role of CGRP in spinal fusion.

## 2. Materials and Methods

### 2.1. Animals and Surgical Procedure

20 male SD rats (each of which weighs 300–350 g) were provided by Shanghai Super—B&K Laboratory Animal Corp., Ltd. Animals were bred and maintained in a 27°C constant temperature room. All the experimental procedures were performed under the guidance of the animal experimental ethics inspection of Laboratory Animal Center in Second Military Medical University. The demineralized freeze-dried bone graft was provided by Aorui Biological Material Co., Ltd., Shanxi, China.

The rat was anesthetized in prone position. Maintained anesthesia was performed by isoflurane (0.5%–2%) and oxygen with a coaxial breath cone. Firstly, a dorsolateral incision (~3 cm) over lumbar 4/5 (L4/5) was performed after shaving the surgical area. Then, the transverse process of L4-L5 could be exposed after bluntly splitting the longitudinal back muscles. Once the transverse process of L4/5 was exposed, decortication was performed until errhysis was observed. The demineralized freeze-dried bone allograft was placed in the decorticated fusion area on L4 transverse process. Finally, the skin and fascia were closed and antibiotics were used intramuscularly for three days. The surgical procedures were performed by X-YZ, S-YW, and X-ZW.

Animals were sacrificed on the 7th day (*n* = 5), 14th day (*n* = 5), 21st day (*n* = 5), and 28th day (*n* = 5) separately after fusion surgery for further assessment.

### 2.2. Histologic Analysis

The rats were sacrificed on the 7th, 14th, 21st, and 28th day after operation, respectively (*n* = 5/week). L4-L5 transverse process fusion area was taken and fixed into 4% paraformaldehyde for 24 hours. Then the samples were decalcified by 5% nitric acid for 72 hours. After being washed in distilled water, the samples were embedded in paraffin. Transversal sections (5 *μ*m) were performed along the midline of the fusion area near the transverse process. Sections were stained by hematoxylin and eosin (H&E) and observed with light microscopy.

### 2.3. Immunohistochemical Staining

Immunohistochemical analysis was performed on 4% paraformaldehyde-fixed paraffin-embedded spine tissue immediately after manual palpation. Regions surrounding transverse process and graft were defined as areas of interest. Sections were incubated with goat polyclonal primary antibody of CGRP (diluted at 1 : 3000, Santa Cruz, CA, USA) for 24 hours. Then, fluorescein isothiocyanate conjugated bovine anti-goat secondary antibody (diluted 1 : 1000, Santa Cruz, CA, USA) was applied for 1 hour. The sections were examined by Olympus Eclipse 80i (Olympus, Japan). The area of CGRP was calculated by Image-Pro Plus 5.02 (Media Cybernetics) under microscope with 400x magnification. Density of CGRP was calculated as (CGRP-positive area/total image area in ×400 magnification) × 100%.

### 2.4. Statistical Analysis

The statistical data was analyzed by SPSS 22.0. The densities of CGRP expression across different fusion stages were compared by ANOVA for repeated measurements. The data of the histomorphometric result was expressed by mean ± standard deviation. *P* < 0.05 was considered as statistically significant difference. Differences of density of CGRP were analyzed at different time points.

## 3. Results

### 3.1. Ectopic Osteogenesis

**Figure 1:**
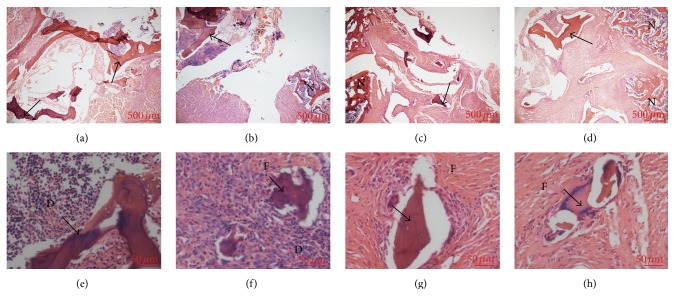
Photomicrographs of the fusion mass on day 7 (a, e), day 14 (b, f), day 21 (c, g), and day 28 (d, h) after spinal fusion surgery. (a), (b), (c), and (d) ×40. (e), (f), (g), and (h) ×400. Black arrows: allograft. F: fibrous tissue. D: deeply stained cell. N: newly formed bone. Bar: 500 *μ*m in ×40 magnification; 50 *μ*m in ×400 magnification.

On the 7th day after surgery, there was no fibrous tissue between the allograft and the transverse process. Only deeply stained cells could be observed in the fusion site. On the 14th day after operation, fibrous tissues could be seen in fusion site. On the 21st day, more fibrous tissues and chondrocytes could be observed and the density of deeply stained cells reduced to a normal level. On the 28th day after spinal fusion surgery, besides the fibrous tissues, new cartilage formed along the ventral side of the allograft with bone marrow cavities (Figure 1).

### 3.2. Spatiotemporal Changes of CGRP-Positive Nerve Fibers during Bone Regeneration

**Figure 2:**
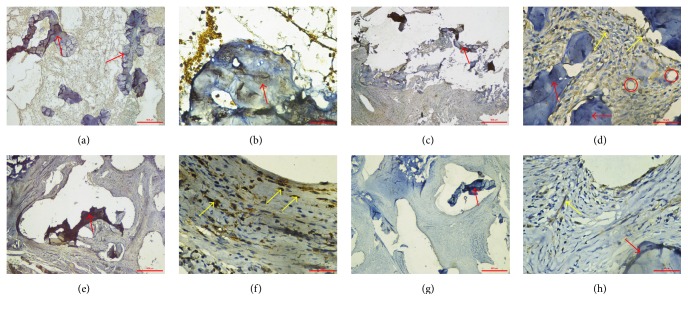
Expression of CGRP on days 7 (a, b), 14 (c, d), 21 (e, f), and 28 (g, h) after surgery. (a), (c), (e), and (g) ×40. (b), (d), (f), and (h) ×400. On the 7th day after operation, nearly no CGRP expression was observed in fusion area. On the 14th day after operation, fibrous tissue formed contacting the allograft and transverse process. CGRP expression could be observed in fibrous tissue and surrounding the vessels in the fusion site. On the 21st day after operation, the expression of CGRP reached peak and most of the CGRP was distributed surrounding the allograft and vessels in fibrous tissue as well as in cartilage. On the 28th day after operation, density of CGRP dropped back into a relatively low level. CGRP expression allocated mainly surrounding the allograft and in the bone meshwork. Red arrows: allograft. Yellow arrows: CGRP expression. Red circles: microvessel. Bar: 500 *μ*m in ×40 magnification; 50 *μ*m in ×400 magnification.

As shown in Figure 2, on the 7th day after operation, nearly no fibrous tissue was formed in fusion site. During the days from 14 to 21, CGRP-positive nerve fiber was always found in the fibrous tissue and surrounding the newly formed microvessels. Particularly, CGRP was mainly expressed surrounding the allograft and vessels in fibrous tissue as well as in cartilage. On the 28th day after surgery, the level of CGRP was reduced but still could be found around chondrocytes.

**Figure 3:**
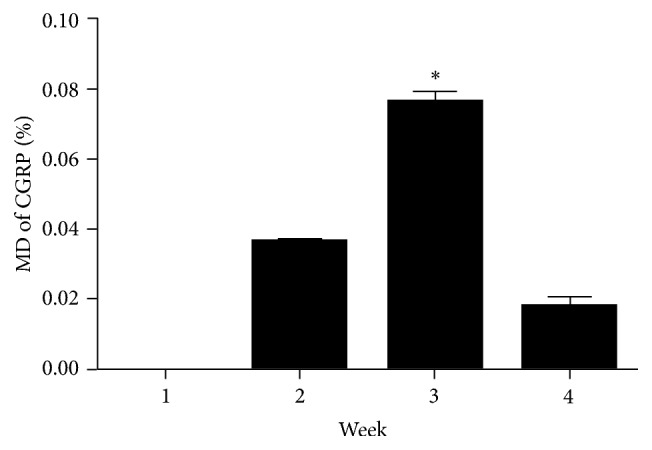
Density of CGRP was significantly higher on the 21st day after spinal fusion surgery compared to other time points. ^*∗*^ *P* < 0.05.

Temporally, on day 7, no CGRP expression was observed in fusion site. In the following two weeks, CGRP-positive nerve fibers increased markedly in fibrous tissue and surrounding the allograft. The density of CGRP reached peak on the 21st day after operation, which is 7.64 ± 0.08% of the fusion site (Table 1 and Figure 3). On day 28, the density of CGRP dropped back into a relatively low level at 1.88 ± 0.04% of the fusion site. According to the results of one-way ANOVA, the density of CGRP in week 3 was higher than that in the other weeks (*P* < 0.05).

## 4. Discussion

In the present study, the spatiotemporal changes of CGRP were investigated during spinal fusion to explore the role CGRP plays in bone regeneration. The results demonstrated that the early stage of normal spinal fusion is accompanied with the increase of CGRP, indicating the potential role of CGRP during spinal fusion.

Previous reports have indicated that CGRP, with the ability to increase 30~50-fold osteoblast cyclic adenosine monophosphate (cAMP), can directly act on osteoblasts [12]. The study of Lam et al. demonstrated that transgenic mice overexpressing CGRP in osteoblasts had significantly increased bone formation rate and bone volume. They revealed that CGRP influence bone metabolism not only through nervous system but also through an autocrine loop through osteoblasts [11].

The spatial change of CGRP-positive nerve fibers was investigated in the present study. Evidence indicated that CGRP mainly exists in bone through sensory nerve fibers, especially in epiphyseal trabecular bones [13]. In the present study, we found that CGRP-positive nerve fibers existed closely around fibrous tissues, vessels, and allograft at fusion site, where mesenchymal cells differentiated into chondrocytes and osteoblasts. The results demonstrated that CGRP-positive nerve fibers mainly located at the tissue-bone surface on the 21st day after operation, when CGRP expression reached peak. This interesting phenomenon suggested that CGRP might have direct effects on old bone absorption and new bone formation during spinal fusion. In* in vitro* studies, He et al. used primary osteoblasts obtained from newborn rabbit calvaria and different concentrations of human CGRP to investigate the role of CGRP in bone metabolism. They found that CGRP elevated the expression of cAMP, ATF4, and OPG, while downregulating the expression of RANKL in a dose-dependent manner, indicating CGRP might stimulate osteoblast differentiation and inhibit osteoclast formation [8]. In addition, Wang et al. found that CGRP-positive nerve fibers were distributed in newly formed cartilage, bone, and fibrous tissues as well as in bone marrow during bone fracture healing [14]. These studies supported our speculation that CGRP might promote spinal fusion through regulating bone formation and absorption. As for the CGRP expression surrounding vessels, there is evidence showing that CGRP-positive nerve fibers are widely distributed in the vascular system [15]. As CGRP is one of the most potent vascular dilators known up to now, it may have an influence on spinal fusion via promoting angiogenesis and newly formed tissue perfusion.

Temporally, the density of CGRP in fusion site can be observed on the 14th day after spinal fusion surgery. During bone fracture healing, hematoma stage occurs at approximately 6–8 hours after injury. In the next 3-4 weeks, hematoma is gradually absorbed and replaced by fibrous tissue [16]. The ingrowth of newly formed vessel occurs at primary callus stage at approximately week 3 after injury [17]. The study carried out by Wang et al. revealed that CGRP-positive nerve fibers remained at a relative low level at week 1 and reached peak density at week 3 [14]. The present study demonstrated that CGRP-positive nerve fibers could be observed on day 7 and continued on a high level until day 21, suggesting that CGRP potentially plays a role in promoting hematoma absorption, fibrous tissue formation, and angiogenesis. Another interesting phenomenon of the present study is that CGRP-positive nerve fiber was not obviously observed on the 7th day after spinal fusion surgery. Since the first week after surgery is mainly the inflammatory phase, we speculated that CGRP might not be involved in the inflammation phase of bone regeneration. On the 28th day after operation, the density of CGRP significantly decreased to a relatively low level but still could be observed around chondrocytes. An explanation might be that CGRP have effects on bone absorption and formation especially endochondral ossification, but not in the bone remodeling phase.

Based on the spatiotemporal changes of CGRP and location during spinal fusion, we noted that on day 14 CGRP mostly located in soft fibrous tissues and surrounding microvessels. On the 21st day after operation, the highest CGRP expression was presented on the interface of allograft and fibrous tissue which would later become newly formed bone. These findings suggested that the increase of CGRP is probably regulated by signaling pathways related to osteogenesis and bone absorption. The assumption can be supported by Li et al.'s study that the density of CGRP in concave side was higher than that in the convex side in an angulated fracture model [18].

There are several limitations in the presents study. Firstly, the spine in a rat is too small to measure the size of the fusion mass. Secondly, the allocation of CGRP is observed in cross sections, which makes it difficult to restore the accurate distribution of CGRP in fusion site in a 3D vision. Thirdly, the mechanism by which CGRP regulate spinal fusion was not investigated in the present study. Further studies may focus on the regulatory function of CGRP during the process of spinal fusion.

## 5. Conclusion

Our study investigated the temporal and spatial changes of CGRP during the normal process of spinal fusion. Furthermore, the density of CGRP reached peak on the 21st day after surgery and most of the CGRP expression located surrounding the interface of allograft and fibrous tissue. These observations of the present study indicated that CGRP might be involved in the process of bone formation and absorption and have an influence on bone metabolism.

## Figures and Tables

**Table 1:** Mean Density (MD) of CGRP expression in fusion area.

Day	7	14	21	28
MD of CGRP (%)	0	3.69 ± 0.03	7.64 ± 0.08^*∗*^	1.88 ± 0.04

Values indicate mean ± standard deviation. ^*∗*^Significant difference (*P* < 0.05).
